# Interprofessional Collaboration in the Detection of and Early Intervention in Child Maltreatment: Employees' Experiences

**DOI:** 10.1155/2013/186414

**Published:** 2013-04-08

**Authors:** Jaana Inkilä, Aune Flinck, Tiina Luukkaala, Päivi Åstedt-Kurki, Eija Paavilainen

**Affiliations:** ^1^School of Health Sciences (Nursing Science), University of Tampere, FI-33014 Tampere, Finland; ^2^Hospital District of Helsinki and Uusimaa, Hyvinkää Hospital, Sairaalankatu 1, FI-05850 Hyvinkää, Finland; ^3^National Institute for Health and Welfare, P.O. Box 30, FI-00271 Helsinki, Finland; ^4^Science Center, Pirkanmaa Hospital District, School of Health Sciences, University of Tampere, FI-33014 Tampere, Finland; ^5^Pirkanmaa Hospital District, P.O. Box 2000, FI-33521 Tampere, Finland; ^6^Southern Ostrobothnia Hospital District, Huhtalantie 54, FI-60220 Seinäjoki, Finland

## Abstract

Child maltreatment is a global problem and a multidimensional phenomenon occurring in all social classes. This study depicts interprofessional collaboration associated with the detection of and early intervention in child maltreatment taking place in the family. The data were collected in a large Finnish city, Tampere (207 866 citizens). A survey was administered to employees in day care, basic education, social and health services, and police (*n* = 865). The results indicate that interprofessional collaboration associated with the detection of and intervention in child maltreatment was best accomplished by social service employees and police personnel. Employees in day care, basic education, health services, and police had little knowledge of the methods used in other units. The most support for collaboration was reported by employees in social services and day care. The results provide basic knowledge of interprofessional collaboration associated with child maltreatment between the agencies involved in the study. The research evidence can also be utilized in an international context when developing collaboration between different fields.

## 1. Introduction

The United Nations Convention on the Rights of the Child [1] emphasizes the best interests of the child and his or her right to special protection. The convention is an effort to secure the child's right to parents and family, but on the other hand, emphasis is laid on society's obligation to offer the child protection and care if the child is being maltreated. Following the age definition laid out in the convention, the present study defined all human beings under the age of 18 as children.

In this study, child maltreatment refers to physical and psychological abuse, sexual abuse and neglect occurring in the family, and living in the atmosphere of domestic violence. Different forms of maltreatment may appear either in isolation or in various combinations, and it is often difficult to make a distinction between the different forms of abuse [2–5]. Child maltreatment may vary in severity: it can involve any activity or lack of activity associated with a child or a child's life circumstances resulting in a deterioration of the child's life situation [2–4].

Child maltreatment is present in all societies, but due to different methods of recording statistics and differences in the detection of the phenomenon, there are no reliable and comparable figures on the incidence of child maltreatment. National assessments and comparisons in Western countries have been made, however [4]. In addition, comparison is made difficult by the fact that corporal punishment of children is not prohibited by law in all countries. According to cross-national estimates, approximately one in ten of child maltreatment cases make their way into official statistics. For instance, in the United States even as many as 900,000 children annually are estimated to be victims of abuse [5, 6]. In the Western countries, approximately 4–16 percent of children are exposed to physical abuse annually, and one in ten children are neglected or exposed to psychological abuse. Five to ten percent of girls and five percent of boys have experienced childhood sexual abuse [4]. A Finnish study of victims of abuse revealed that 12 percent of children under 15 had been victims of mild domestic abuse during 2008, while 4 percent had been victims of serious abuse [7]. Twelve percent of children had witnessed domestic violence between parents [8]. In Finland, mild domestic abuse experienced by children has dropped substantially over the past 20 years, but the levels of severe domestic abuse have remained almost unchanged. Instances of father-daughter incest were reported by 0.2 percent of girls while step-father-daughter incest was reported by 2 percent of girls. Boys did not report experiences of incest [7].

The detection of and intervention in child maltreatment are always difficult and challenging tasks to achieve for professionals working with children. In earlier research, it has been shown that the detection of and early intervention in child maltreatment call for interprofessional collaboration [2, 9] to pool the knowledge, competencies, and resources of employees. In addition, separate agencies, workplace cultures and tasks require competence in interprofessional collaboration [10–14]. Interprofessional services for children and families related to the detection of and intervention in child maltreatment are organized with different methods on both the national and international level. When collaboration takes place across professional and organizational boundaries and work units, the different views, tasks, and responsibilities interfere with interprofessional work, even though they share the concern for a child's situation. In the development of special competence for different professions and collaborational skills, the central goals are to increase knowledge and identify both common work practices among different professions and practices that are specific to each profession [15].

In this study, interprofessional collaboration refers to collaboration and teamwork between the employees of different organizations, agencies and units, and professional groups. The partners in interprofessional collaborations are all professionals coming into contact with families of children, with each professional group having its own important role [2, 3, 9]. The focus is on employees who meet children everyday and who encounter situations where there is reason to suspect and evaluate the possibility of maltreatment [11, 16]. In order to promote collaboration, the support of the superiors [17] and the work community is vital [11]. The collaboration is based on the needs and safety of children [9, 13].

According to a report by Unicef [18] on the well-being of European children, more information is required on domestic violence experienced by children. Child maltreatment and interprofessional collaboration have been in the focus of researches for decades, but at the same time these themes have rarely been combined under the same study. In most studies, the focus has been on examining the collaboration between a few professions and work units (e.g., [19]). In addition, the content of the terms used in studies has varied. The term *child maltreatment* has been used to refer to violence experienced by children and youth (e.g., [16, 20, 21]). The terms *child abuse* or *violence experienced by children* have been used as parallel terms to child maltreatment [2, 22, 23]. Earlier studies have focused on signs of physical abuse, reasons behind violent behavior, and individual events from the point of view of the victims of violence and perpetrators of violence. Child sexual abuse has been studied from the point of view of physical findings and symptoms. It is more difficult to give evidence of emotional abuse and neglect than of physical abuse, and therefore it is identified and studied less. *Maltreatment* is an umbrella term that covers both negative actions and neglected actions towards a child [3, 24]. The information obtained is scattered and it does not give a clear holistic picture of the interprofessional collaboration between several actors that is related to detecting and intervening in child maltreatment. Therefore, it is problematic to utilize results from earlier studies in this study. It is important to study the topic in a broad context, with a large sample size and simultaneously from the points of view of employees in several different professions [16, 25].

This study is part of a larger research project, “detection and treatment of domestic abuse,” undertaken in the Department of Nursing Science at the University of Tampere and funded by the Academy of Finland (no. 109830, 2006–2008). The goal of this study is to produce information for developing the practices of several collaborational actors in the detection of and intervention in child maltreatment. The purpose of the study was to describe (1) employee competence in interprofessional collaboration, (2) how the perspectives of other agencies have been taken into account, and (3) the support received for collaboration in the detection of and early intervention in child maltreatment within the family.

## 2. Methods

### 2.1. Instrument Development

The development of the instrument drew on the literature search conducted using the Linda, Medic, Cinahl, Medline, Psychinfo, EBM reviews, and British Nursing Index databases over the years 2000–2007. The UN Convention on the Rights of the Child [1] was also used as a basis for the development.

**Figure 1:**
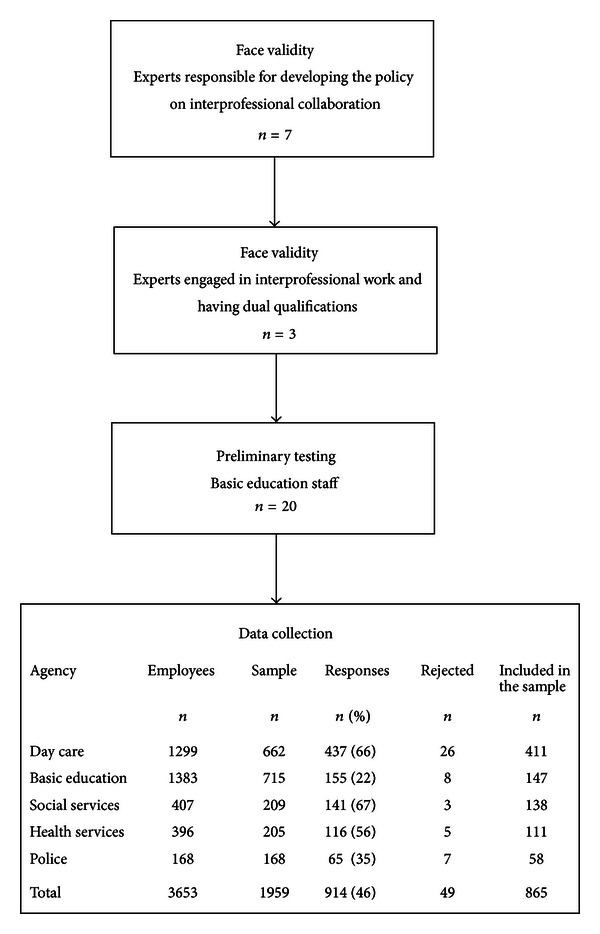
Scale development and data collection by fields (*n* = 865).

The instrument was first assessed by content experts (*n* = 7) who have developed collaboration related to the detection of and intervention in child maltreatment. The instrument, revised based on the expert appraisal, was then evaluated by experts (*n* = 3) with two professional qualifications working in different agencies. Pretesting was carried out with employees (*n* = 20) from one basic school within (Figure 1).

The scale consists of an eight-page questionnaire, where the domains of collaboration included the competence of the employees in interprofessional collaboration, taking the perspectives of other collaboration agencies into account, and support for collaboration [2, 9, 16, 26]. The attitude statements were rated on a 6-point Likert scale (1 = definitely disagree, 2 = disagree, 3 = somewhat disagree, 4 = somewhat agree, 5 = agree, 6 = definitely agree).

Research permissions were obtained from the directors of day care, basic education services, and police in the city of Tampere and from the Research Permission Committee of health and social services. Ethical approval for the study was obtained from the Pirkanmaa Hospital District Ethics Committee (R07019H).

Tampere is the third largest city in Finland with a population of 207 866 in 2007. There were 35 629 youth and children under 18 years of age, which is 17% of the whole population. There were altogether 20 588 families with children in Tampere [27].

### 2.2. Data Collection

The basic group in the study consists of day care, basic education, social services, health care, and police personnel in the city of Tampere. The data were collected by collecting a stratified sample of 50% from units that work with children under the age of 18. A census of police officers was conducted because of the small size of the group [28]. The total sample consisted of 1 959 employees. The line managers of the work units distributed the survey forms to the personnel so that they would represent a diverse group of employees in the work community, including all professions, different sexes, different ages, different lengths of work experience, and both permanent and temporary workers. The data were collected during 15.3–13.9 in 2007. The survey forms were returned to the researcher in a closed envelope. A total of 914 questionnaires were received, giving a response rate of 46%. Forty-nine questionnaires were rejected because of missing data, as over 20% of the responses to a set of items were missing. As shown by the dropout analysis, the background characteristics of those who returned an incomplete questionnaire did not differ substantially from those included in the study. The survey achieved the principle of representativeness for all units. According to the loss analysis, the questionnaire achieved the principle of representativeness for all work units (Figure 1).

### 2.3. Statistical Analysis

A statistical analysis was performed using SPSS for Windows 18.0. If a questionnaire had less than 20% missing values (*n* = 16), these were replaced by the mean of scores on collaboration variables.

A principal component analysis (PCA) was conducted in order to find out which statements measure similar properties. PCA showed that all items (*n* = 13) correlated with at least one item (*r* > 0.30). The number of principal components was limited to components with the eigenvalue of over one and with the variation of the explanation parts exceeding 5%. However, the large sample size may have caused the statistical significance. Nevertheless, the principal component analysis calculated the Pearson correlation coefficients for skewed distributions and for statements with ordinal scales, which may weaken the analysis. Items loading heavily (>0.40) on a component were included in the scale. The number of the principal components was tested with free and forced factoring, and the results were similar. As a result of an oblique promax rotation, the variables were loaded to only one factor and their content was meaningful. Considering the subject of study, it is significant that the statements can correlate with each other. The commonalities of individual items ranged from 0.437 to 0.777, demonstrating that the variables measured the principal components fairly reliably [28].

Three principal components based on previous theoretical knowledge emerged. These were given names according to the item content: competence in interprofessional collaboration (5 items), taking into account the perspectives of other collaboration agencies (3 items), and receiving support for collaboration (5 items). The three principal components accounted for 59% of the total variance (Table 1).

With the principal component analysis, the summated scales were formed in order to examine the phenomenon holistically. Three summated scales were formed by adding up the items depicting each principal component and by dividing the sum by the number of items. This made the summated scales mutually comparable, although the number of items varied [28].

The internal consistency of the sum variables was examined by using Cronbach's alpha values. The values ranged from 0.602 to 0.830, and the total alpha value of 0.840 demonstrated that the instrument was internally consistent [28] (Table 1).

The distributions of respondent characteristics (gender, age, education, work experience at the present unit, total work experience, and employment status) were described using frequencies and percentages. The distributions of the three summated scales formed by using the principal component analysis for crosstabulation were reclassified into two classes (disagree = 1.0–3.4, agree = 3.5–6.0). This solution was supported by the fact that the observations fell into all of the categories. In the results section, the distributions of the items are described using the percentages of those who agreed and disagreed with an item. Additionally, we present the frequencies and percentages of those agreeing with an item by agencies in Table 3 because there were statistically highly significant associations between the agencies and the items. The associations between background information and the items were examined by using crosstabulation analysis, chi-square analysis, or Fisher's exact test, if the expected frequencies were too small. The significance level was set at <0.01 due to the large size of the data set [28].

## 3. Results

### 3.1. Demographic Characteristics of the Respondents

The majority of the respondents were women. The age range of respondents was from 20 to 64 years, with a mean age of 43 years. The average amount of work experience was 15 years (range 1 month–42 years) (Table 2). Forty-eight percent of the respondents worked in day care, 17% worked in basic education, 16% in social service, 13% in health service, and 7% in police departments (Figure 1). 

### 3.2. Competence in Interprofessional Collaboration

The majority of the respondents (93%) had the competence to collaborate with other officials. The lowest ratings of their competence were given by health service employees (*P* = 0.002, *ϕ* = 0.140) (Table 3).

Eighty-two per cent knew what to do when collaborating with other agencies to detect child maltreatment. However, employees in day care and basic education evaluated their collaborative competence as the weakest (*P* < 0.001, *ϕ* = 0.180) (Table 3). Eighty-four per cent of permanent employees and 79% of contract employees knew how to collaborate with other agencies (*P* = 0.002, *ϕ* = 0.113).

Three-fourths (74%) of the employees knew how to act when detecting child maltreatment. It is noteworthy that one-third of the employees in day care, basic education, and health services did not know what to do when detecting child maltreatment (*P* < 0.001, *ϕ* = 0.167) (Table 3). Young respondents had the least knowledge and skills (*P* = 0.006, *ϕ* = 0.116). Seventy-seven per cent of permanent employees and 64% of contract employees knew how to act (*P* = 0.001, *ϕ* = 0.121).

Sixty-eight per cent of the respondents were also capable of acting independently when intervening in child maltreatment. The highest ratings of independent action in child maltreatment cases were given by social service employees and police officers (*P* < 0.001, *ϕ* = 0.229) (Table 3). Employees were better able to act independently as they grew older (*P* = 0.004, *ϕ* = 0.129). Eighty per cent of men and 66% of women (*P* = 0.004, *ϕ* = 0.097) and seventy-one per cent of permanent staff and 57% of contract staff were capable of acting independently (*P* = 0.001, *ϕ* = 0.129).

Half of the respondents (50%) had knowledge of the practices of others; health care employees had the least knowledge of the practices of others (*P* < 0.001, *ϕ* = 0.171).

### 3.3. Taking into Account the Perspectives of Other Agencies in Collaboration

Of those who had five years work experience or less, 2% did not appreciate the competencies of employees in other agencies (*P* = 0.006, *ϕ* = 0.124).

Women (96%) were more accepting than men (90%) of the various perspectives (*P* = 0.001, *ϕ* = 0.109) of other agencies, whereas police officers were the least accepting (*P* = 0.002, *ϕ* = 0.142).

### 3.4. Receiving Support for Collaboration

The majority (81%) of the respondents received support from other agencies in child maltreatment detection. However, one-third of those working in basic education and health services did not receive support from other agencies (*P* < 0.001, *ϕ* = 0.162) (Table 3). Those who had a Bachelor's-level degree received the least support (*P* = 0.001, *ϕ* = 0.136).

The vast majority (90%) of the respondents received support from their own unit to detect child maltreatment. It is noteworthy that 30% of police officers did not receive support from their unit for detecting child maltreatment (*P* < 0.001, *ϕ* = 0.275) (Table 3). The age of the respondent (*P* = 0.001, *ϕ* = 0.137), total work experience (*P* = 0.009, *ϕ* = 0.119), and work experience from the present unit (*P* = 0.001, *ϕ* = 0.146) increased support. In addition, the more time had elapsed after graduation, the more support the respondent received (*P* = 0.003, *ϕ* = 0.128). Nineteen per cent of men and 8% of women did not receive support from their unit (*P* < 0.001, *ϕ* = 0.124). Those who spent less than half their working day (76%) working with children received less support than those who spent the entire day (94%) working with children (*P* < 0.001, *ϕ* = 0.228).

Nearly all (94%) employees received support from their supervisors for interagency collaboration associated with child maltreatment. Health service employees did not receive support from the supervisor for interagency collaboration (*P* < 0.001, *ϕ* = 0.218) (Table 3). Those with a Bachelor's-level degree (89%) received the least support from their supervisor compared with those who had a Master's-level degree (96%) and those with college-level qualifications (96%) (*P* = 0.007, *ϕ* = 0.127). Those who spent less than half their working day (87%) working with children received less support from their supervisor than did those who spent the entire day working (96%) (*P* < 0.001, *ϕ* = 0.145).

The majority (92%) of the respondents were satisfied with the collaboration in their own unit while the highest levels of dissatisfaction were reported by police officers (*P* = 0.001, *ϕ* = 0.149). Those who spent half or more than half their workday (94%) working with children were more likely to be satisfied with the collaboration in their unit than those who spent less than half (86%) their time working with children (*P* = 0.001, *ϕ* = 0.111).

Sixty per cent of the respondents had time for interagency collaboration. Social service employees had the most time for interagency collaboration (*P* < 0.001, *ϕ* = 0.235) (Table 3). Those who spent less than half (47%) of their workday with children had less time for interagency collaboration than those who spent half or more (63%) of their work day with children (*P* < 0.001, *ϕ* = 0.131). 

## 4. Discussion

The purpose of the study was to describe employee competence in interprofessional collaboration, how the perspectives of other agencies have been taken into account, and the support received for collaboration in the detection of and early intervention in child maltreatment within the family.

### 4.1. Main Results

There was a statistically highly significant association between the respondent's field and *competence in interprofessional collaboration*. The highest level of competence in interprofessional collaboration was reported by social service employees and police officers. The result concerning social service employees was as expected since they are the key actors in maltreatment issues [29]. Accordingly, the lowest rating of skills was given by employees in basic education and day care, in other words those who meet children regularly everyday. The employee's individual and positive attitudes and willingness to collaborate are factors that affect interprofessional collaboration [11, 12, 26, 30]. According to the results of a study by Rae et al. [29], the knowledge of health care service workers regarding legislation prohibiting punitive violence varied significantly. Djeddah et al. [2] and Paavilainen and Flinck [3] state that employees in social services and health care should be more active and willing to engage in interprofessional collaboration. Permanent employees were more knowledgeable than contract employees about how to act when detecting child maltreatment. In addition, permanent employees were more independent than contract employees when intervening in child maltreatment. The results may have been affected by the strict secrecy clauses in Finnish health care and uncertainty about the legal possibilities and restrictions on collaboration [31]. Employees can also be required to take personal responsibility and show dedication to collaboration [17, 19]. Goebbels et al. [11], Cerezo and Pons-Salvador [16], and Rae et al. [29] emphasize that information on the detection of and intervention in child maltreatment is important to all employees, regardless of their specialty or level of education. This kind of competence in interprofessional collaboration is related to the effectiveness of collaboration in practice [12].

In this study, only half of those working in day care, basic education, health services, and police were aware of the methods used by other agencies. The result corroborates earlier findings in a study by Afza et al. [10] on health services, showing that the methods used in child welfare services were poorly recognized. In this case, there may be a risk that interprofessional competence is not utilized sufficiently [19]. The results of the present study are relevant, as Cleaver and Walker [9] and Green et al. [12], and Clarke [19] have shown that knowledge of the tasks, responsibilities and methods used by other professionals reduces mistrust and increases interprofessional action. The practical implementation of interprofessional collaboration requires joint discussion and learning both within work communities and between the employees of collaborating organizations [16, 32]. According to earlier studies, child maltreatment was not intervened in systematically and with determination [11], and therefore health care workers need training, change in attitudes, and simple tools for detecting and intervening in child maltreatment [16, 19, 33, 34].

The results agree with the study by Rae et al. [29] regarding the fact that the length of work experience has no effect on knowledge of child protection legislation and practices regarding punitive violence. In a study by Goebbels et al. [11] and McKenzie et al. [35], however, increasing work experience increased knowledge and practical information among health care workers.

There was a statistically highly significant association between the field of employment and *receiving support for collaboration*. Employees need support for the detection of and intervention in child maltreatment [16, 22, 36]. Women employees in social services and day care and employees who spent half or more of their workday with children received the most support. According to Chanmugan [31], all employees need support from the line manager and peer support from the work community and on several occasions, if necessary. The employees mostly emphasized details related to working correctly regarding ethics and legislation. This result agrees with earlier studies in that support from the superior [17] and the work unit [11] had a big influence on the collaboration in the detection of and intervention in child maltreatment. The most common sources of support were the supervisor and the employee's own unit. The amount of time spent working with children was also associated with the receipt of support from the supervisor and the work unit [16, 20]. The result is understandable, as the issues of detecting and intervening in child maltreatment are primarily discussed within the employee's own unit with familiar coworkers [11, 19]. On the other hand, the role of supervisors in the collaboration process should be studied more closely [26].

In this study, one-third of the employees in basic education and health services felt that they did not receive support from other agencies for detecting child maltreatment. The result is worrying because the possibility for consultation across organizational boundaries has been regarded as an important form of support when detecting maltreatment [9, 16, 31]. For example, in the study by Clarke [19], social services were an important collaboration partner for the school nurse.

In addition, the support received depended on legislation, operating practices, government support, and societal decisions [16, 25]. For example, the paediatrics committee on child abuse and neglect of the American Academy [22] recommends approving laws prohibiting the use of punitive violence. Some states now place an act of child abuse on the central registry only when the abuse is considered “serious.” Financial considerations and the legitimate fear of being overwhelmed by the number of abuse children has led some child protective services systems to construct a triage system whereby a child has to be in relatively imminent danger or seriously abused before there will be a response.

The results of this study show that social service employees had the most time for interagency collaboration. Similar results were obtained by Ødegård [30] in a study of children's mental health care where employees used nearly half their working day on collaboration. It is clear that the development of good collaboration calls for time and discussions [12, 14, 25, 26, 37].

In this study, especially separate agencies and fields of employment emerged as hindrances to collaboration. The lack of shared models of thought and the small amount of collaboration may have affected the views of the respondents. In addition, different work tasks and professional insecurity in collaboration related to detecting and intervening in child maltreatment may have had an effect on the results, especially in the responses of employees in day care, basic education and health care. According to the results of a study by Bunting et al. [37], in addition to joint interprofessional training, open and common discussion and sharing of experiences regarding interprofessional collaboration, as well as information on the job descriptions of collaborating partners, is needed to increase joint activities and efficiency. In difficult situations feelings and responsibility can be shared with others. Through interprofessional training and collaboration, employees would get support for detecting and intervening in child maltreatment. At minimum, the training should include the detection of signs of abuse, and when how, and where should abuse be reported [16, 20]. It would be useful to offer training on different occasions as basic, continued, and complementary training [37].

### 4.2. Reliability of the Study

Content validity was strengthened in a stepwise manner by expert assessments and preliminary testing. The alpha values for the summated scales ranged from 0.602 to 0.830. Alpha values of ≥0.70 can be considered relatively good for a new instrument. The summated scale “Taking into account the perspectives of other agencies” contained only three items, which may have contributed to the low alpha value [28]. 

Within a large sample, even small differences between groups or associations between variables can be statistically significant as determined by the chi-square analysis. Based on an assessment, a marked content-related association is called significant [28]. In this study, there was a significant association between the agencies and the items.

External validity was assessed on the basis of the sample, response rate, and representativeness of the data [28]. To increase the reliability of the study, the data were collected by drawing a stratified sample of 50% from each unit because the response rate was estimated to vary. The response rate (46%) may have been reduced by the employees' perception of child maltreatment detection as not being the core task of their work, although the child welfare act [38] stipulates that child maltreatment detection is the responsibility of all employees. The comments given in the questionnaires mentioned the difficulty of the research topic as the reason for missing data. The scale did not include the response options “cannot say” or “does not concern me,” which may have contributed to the fact that some respondents returned a blank or partially completed questionnaire. Because of the missing data, altogether 49 questionnaires were rejected.

The number of respondents was large with regard to the target population. The sample size should be 10 per cent of the survey population to allow generalization of results [39]. The respondents (*n* = 856) represent 24% of all employees. The survey achieved the principle of representativeness for the agencies as the following percentages show: day care 24%, basic education 11%, social services 34%, health services 28%, and police 34%. The results of the study represent the study sample. This study does not give an explanation for the low response rates from basic education and health care services, but it does raise several further questions and present a topic for further research. The response rate may have been affected by the different backgrounds of workplace cultures and traditions in the respective lines of activities, as well as the professional role and the connection of the respondents' core work tasks to child maltreatment. In the studies of Marijcke and Browne [23] and Safeguarding Children [13], the employees did not have the readiness, skills, and means to intervene in child maltreatment. Collaboration related to the detection of and intervention in child maltreatment usually begins when an employee becomes concerned and suspects maltreatment or when a family member reports it [11]. Basic education and health care services are central everyday environments in a child's life where it is possible to identify maltreatment [3, 11, 16]. 

The comments led us to assume that employees in different fields of employment considered the research topic important. A number of questionnaires included a comment that the respondent does not have experience of detecting or intervening in child maltreatment, so their responses were based on an assumption of how they would act. This decreases the reliability of the study, as respondents may have responded in a socially desirable way [28]. In general, the respondents evaluated themselves as competent, but differences emerged between different agencies. The respondents were critical of how they would act, and therefore the use of self-evaluation is not a major problem.

## 5. Conclusions

We can conclude that the employees rated interprofessional collaboration fairly positively. The result corroborates the results of previous national and international studies in that the detection of and intervention in child maltreatment require employees to have competencies in interprofessional collaboration that transcend organizational boundaries. These results point to the need to develop interprofessional collaborative competencies for detecting child maltreatment especially among employees in day care, basic education, and health services, because these are the professionals that work with children on a regular basis. 

More attention needs to be paid to hands-on collaboration. These results call for increased collaboration between different agencies, for example, by using mutual visits to get to know and better understand the work of others and to make contacts easier. Through regular meetings the common goal and aim can be crystallized for employees attending to the same families but working in different agencies/organizations. This could increase the dedication of the employees and increase their appreciation for and knowledge of the work of the other agencies.

### 5.1. Implications for Practice

The results can be utilized in developing and implementing shared training for different professions. It is possible to develop practical collaboration through interprofessional training. The training should focus on familiarizing the employees with the job descriptions of employees in other agencies/organizations and on discussing what interprofessional collaboration means to each individual employee and to different professions. The training should discuss what the perquisites of interprofessional collaboration are and how it could be promoted.

A challenge for future research is deepening the knowledge on the topic by interviewing the employees, especially related to differences between the different lines of activities and low response rates. It would also be important to gain knowledge of the experiences of children and families. Using different research approaches and data collected from different agencies, it is possible to get a more versatile picture of the detection of and intervention in child maltreatment than by using only one research method. Further development of collaboration will benefit children and their families, as maltreatment will be detected at an early stage.

## Figures and Tables

**Table 1:** Competence in interprofessional collaboration, consideration for the perspectives of other agencies, and receiving support for collaboration: commonalities and percentages accounted for by the principal components, and the internal consistency of the scale as assessed by Cronbach's alpha (*n* = 865).

Principal component	Commonality	Percentage accounted for %	Number of items	Cronbach's alpha
Competence in interprofessional collaboration	0.437–0.777	35	5	0.830
Consideration for perspectives of other collaboration agencies	0.484–0.663	15	3	0.602
Receiving support for collaboration	0.452–0.686	9	5	0.791

All items	0.437–0.777	59	13	0.840

**Table 2:** Respondent background characteristics and job-related background factors (*n* = 865).

Background factors	*n*	(%)
Gender		
Female	747	(86)
Male	117	(14)
Missing data	1	(0.1)
Age in years		
<30	115	(13)
30–39	217	(25)
40–49	285	(33)
≥50	243	(28)
Missing data	5	(0.6)
Education		
No vocational qualifications	3	(0.3)
College-level vocational qualifications	288	(33)
Bachelor-level (postsecondary level/polytechnic degree)	182	(21)
Master-level (undergraduate/postgraduate degree)	385	(45)
Missing data	7	(0.7)
Work experience at the present unit in years		
≤1	147	(17)
2–4	182	(21)
5–10	195	(23)
11–20	164	(19)
≥21	157	(18)
Missing data	20	(2.3)
Total work experience in years		
≤5	160	(19)
6–15	282	(33)
16–25	230	(27)
≥26	145	(17)
Missing data	48	(5.5)
Employment status		
Permanent	684	(79)
Contract	173	(20)
Other	6	(0.7)
Missing data	2	(0.2)
Time spent working with children		
Less than half of work day	155	(18)
Half or more than half of work day	700	(81)
Missing data	10	(1.2)

**Table 3:** Employee perceptions of competence in interprofessional collaboration. Percentages of those agreeing with the item. Statistical differences between the fields were tested using the Pearson Chi-square test or Fisher's Exact Test (*n* = 865).

	Day care	Basic education	Social services	Health services	Police		
	*n* = 411	*n* = 147	*n* = 138	*n* = 111	*n* = 58	*P* value	Phi
	%	%	%	%	%		
*Competence in interprofessional collaboration *	*75 *	*76 *	*96 *	*75 *	*90 *	<*0.001 *	
If necessary, I am capable of collaborating with other agencies	90	95	99	89	98	0.002	0.140
I am capable of collaborating with other agencies in child maltreatment issues	78	77	95	81	93	<0.001	0.180
I know what to do when detecting child maltreatment	71	69	89	68	81	<0.001	0.167
I am also capable of acting independently when intervening in child maltreatment	62	63	88	64	86	<0.001	0.229
I am aware of work patterns in other units	47	46	69	41	50	<0.001	0.171

*Consideration for the perspectives of other collaboration agencies *	*97 *	*99 *	*96 *	*99 *	*95 *	*0.280 *	
I appreciate the competence of other employees in another field	100	100	100	100	98	0.384*¹*	0.074
I accept the different perspectives of other agencies	97	97	93	98	86	0.002	0.142
We have a common approach to collaboration in child maltreatment issues with other agencies	88	85	87	84	85	0.721	0.049

*Receiving support for collaboration *	*95 *	*90 *	*96 *	*80 *	*83 *	<*0.001 *	
I receive support from other agencies for child maltreatment detection	86	71	84	73	81	<0.001	0.162
I receive support from my unit for child maltreatment detection	95	91	96	77	71	<0.001	0.275
My supervisor supports inter-agency collaboration related to child maltreatment	96	95	97	81	95	<0.001	0.218
I am satisfied with the collaboration in my unit	93	93	97	86	83	0.001	0.149
I have time for collaboration with other agencies	64	52	79	42	43	<0.001	0.235

*¹*Fisher's Exact Test.

## References

[2] Djeddah C, Facchin P, Ranzato C, Romer C (2000). Child abuse: current problems and key public health challenges. *Social Science and Medicine*.

[3] Paavilainen E, Flinck A Identification of and intervention in child maltreatment. A clinical practice guideline. http://www.hotus.fi/system/files/Child_maltreatment_identification_ENG.pdf.

[4] Gilbert R, Widom CS, Browne K, Fergusson D, Webb E, Janson S (2009). Burden and consequences of child maltreatment in high-income countries. *The Lancet*.

[5] Tenney-Soeiro R, Wilson C (2004). An update on child abuse and neglect. *Current Opinion in Pediatrics*.

[6] Greeley C (2009). The future of child maltreatment prevention. *Pediatrics*.

[7] Ellonen N, Kääriäinen J, Salmi V, Sariola H Violence against children and adolescents in Finland. http://www.optula.om.fi/44610.htm.

[8] Lepistö, S, Luukkaala T, Paavilainen E (2011). Witnessing and experiencing domestic violence: a descriptive study of adolescents. *Scandinavian Journal of Caring Science*.

[9] Cleaver H, Walker S (2004). From policy to practice: the implementation of a new framework for social work assessments of children and families. *Child & Family Social Work*.

[10] Afza M, Wardle S, Light L (2007). Child protection issues: an audit of general practitioners in a primary care trust. *Child Abuse Review*.

[11] Goebbels AFG, Nicholson JM, Walsh K, De Vries H (2008). Teachers’reporting of suspected child abuse and neglect: behaviour and determinants. *Health Education Research*.

[12] Green BL, Rockhill A, Burrus S (2008). The role of interagency collaboration for substance abusing families involved with child welfare. *Child Welfare*.

[13] Safeguarding children. http://www.safeguardingchildren.co.uk/.

[14] Watkin A, Lindqvist S, Black J, Watts F (2009). Report on the implementation and evaluation of an interprofessional learning programme for inter-agency child protection teams. *Child Abuse Review*.

[15] Xyrichis A, Lowton K (2008). What fosters or prevents interprofessional teamworking in primary and community care? A literature review. *International Journal of Nursing Studies*.

[16] Cerezo MA, Pons-Salvador G (2004). Improving child maltreatment detection systems: a large-scale case study involving health, social services, and school professionals. *Child Abuse and Neglect*.

[17] Molyneux J (2001). Interprofessional teamworking: what makes teams work well?. *Journal of Interprofessional Care*.

[18] Unicef (2007). Child poverty in perspective: an overview of child well-being in rich countries. *Report Card*.

[19] Clarke ML (2000). Out of the wilderness and into the fold: the school nurse and child protection. *Child Abuse Review*.

[20] Little L, Kantor GK (2002). Using ecological theory to understand intimate partner violence and child maltreatment. *Journal of Community Health Nursing*.

[21] Gaffney KF, Barndt-Maglio B, Myers S, Kollar SJ (2002). Early clinical assessment for harsh child discipline strategies. *The American Journal of Maternal Child Nursing*.

[22] American Academy of Pediatrics Committee on Child Abuse and Neglect (2002). When inflicted skin injuries constitute child abuse. *Pediatrics*.

[23] Marijcke WM, Browne KD (2003). Identifying abused children using assessments and observations in the classroom: a preliminary study. *Child Abuse Review*.

[24] McAllister M (2000). Domestic violence: a life-span approach to assessment and intervention. *Primary Care Practice*.

[25] Banks D, Hazen AL, Coben JH, Wang K, Griffith JD (2009). Collaboration between child welfare agencies and domestic violence service providers: relationship with child welfare policies and practices for addressing domestic violence. *Children and Youth Services Review*.

[26] D’Amour D, Ferrada-Videla M, Rodriguez LSM, Beaulieu MD (2005). The conceptual basis for interprofessional collaboration: core concepts and theoretical frameworks. *Journal of Interprofessional Care*.

[27] Statistical Finland. http://www.stat.fi/til/index.html.

[28] Burns N, Grove S (2005). *The Practice of Nursing Research. Conduct, Critique and Utilization*.

[29] Rae H, McKenzie K, Murray G (2010). Health care workers’ knowledge of current child protection legislation and child discipline practices. *Child Abuse Review*.

[30] Ødegård A (2007). Time used on interprofessional collaboration in child mental health care. *Journal of Interprofessional Care*.

[31] Chanmugan AA (2009). Qualitative study of school social workers' clinical and professional relationships when reporting child maltreatment. *Children & Schools*.

[32] Morrison T (2010). The strategic leadership of complex practice: opportunities and challenges. *Child Abuse Review*.

[33] Sanders T, Cobley C (2005). Identifying non-accidental injury in children presenting to A&E departments: an overview of the literature. *Accident and Emergency Nursing*.

[34] Ziegler DS, Sammut J, Piper AC (2005). Assessment and follow-up of suspected child abuse in preschool children with fractures seen in a general hospital emergency department. *Journal of Paediatrics and Child Health*.

[35] McKenzie K, Powell H, McGregor L (2004). The impact of control and restraint training on nursing students. *Learning Disability Practice*.

[36] HM Government Working together to safeguard children: a guide to inter-agency working to safeguard and promote the welfare of children. https://www.education.gov.uk/publications/eOrderingDownload/00305-2010DOM-EN.pdf.

[37] Bunting L, Lazenbatt A, Wallace I (2010). Information sharing and reporting systems in the UK and Ireland: professional barriers to reporting child maltreatment concerns. *Child Abuse Review*.

[39] Holm K, Llewellyn JG (1986). *Nursing Research for Nursing Practice*.

